# Gene encoding the CTP synthetase as an appropriate molecular tool for identification and phylogenetic study of the family *Bifidobacteriaceae*

**DOI:** 10.1002/mbo3.579

**Published:** 2018-01-22

**Authors:** Jiří Killer, Chahrazed Mekadim, Radko Pechar, Věra Bunešová, Jakub Mrázek, Eva Vlková

**Affiliations:** ^1^ Institute of Animal Physiology and Genetics of the Czech Academy of Sciences Prague 4 – Krč Czechia; ^2^ Faculty of Agrobiology, Food and Natural Resources Department of Microbiology, Nutrition and Dietetics Czech University of Life Sciences Prague 6 – Suchdol Czechia

**Keywords:** *Bifidobacteriaceae*, *Bifidobacterium*, classification, CTP synthetase, phylogenetics, scardovia

## Abstract

An alternative molecular marker with respect to the 16S rRNA gene demonstrating better identification and phylogenetic parameters has not been designed for the whole *Bifidobacteriaceae* family, which includes the genus *Bifidobacterium* and scardovial genera. Therefore, the aim of the study was to find such a gene in available genomic sequences, suggest appropriate means and conditions for asmplification and sequencing of the desired region of the selected gene in various strains of the bacterial family and verify the importance in classification and phylogeny. Specific primers flanking the variable region (~800 pb) within the *pyrG* gene encoding the CTP synthetase were designed by means of gene sequences retrieved from the genomes of strains belonging to the family *Bifidobacteriaceae*. The functionality and specificity of the primers were subsequently tested on the wild (7) and type strains of bifidobacteria (36) and scardovia (7). Comparative and phylogenetic studies based on obtained sequences revealed actual significance in classification and phylogeny of the *Bifidobacteriaceae* family. Gene statistics (percentages of mean sequence similarities and identical sites, mean number of nucleotide differences, *P*‐ and *K*‐distances) and phylogenetic analyses (congruence between tree topologies, percentages of bootstrap values >50 and 70%) indicate that the *pyrG* gene represents an alternative identification and phylogenetic marker exhibiting higher discriminatory power among strains, (sub)species, and genera than the 16S rRNA gene. Sequences of the particular gene fragment, simply achieved through specific primers, enable more precisely to classify and evaluate phylogeny of the family *Bifidobacteriaceae* including, with some exceptions, health‐promoting probiotic bacteria.

## 1 INTRODUCTION

At present, the *Bifidobacteriaceae* family, classified into the *Actinobacteria* phylum, consists of nine genera: *Bifidobacterium*,* Aeriscardovia*,* Alloscardovia*,* Bombiscardovia*,* Gardnerella*,* Neoscardovia*,* Parascardovia*,* Pseudoscardovia*, and *Scardovia* encompassing 69 (sub)species (http://www.bacterio.net/-classifphyla.html#bifidobacterium). Very recently, the new genus *Galliscardovia* from the crop of a laying hen, new species *Bifidobacterium apri* from the digestive tract f wild pigs and *Alloscardovia venturai* from the oral cavity of a guinea‐pig were described (Pechar et al., 2017b,2017c; Sechovcová et al., 2017). All members of the family have a specific activity of fructose‐6‐phosphate phosphoketolase (F6PPK) that distinguishes them from other bacteria (Bunesova, Vlkova, Rada, Killer, & Musilova, 2014; Killer et al., 2010). Nevertheless, F6PPK has very recently been proven in the order Coriobacteriales, thus saccharolytic bacteria inhabit a similar ecological niche to bifidobacteria. Horizontal transfer between Coriobacteriales and bifidobacteria was hypothesized (Gupta, Nanda, & Khadka, 2017).

Bifidobacteria primarily inhabit the intestines of mammals. However, their presence was also proven in the oral cavity of mammals, human urinary tract and vaginal environment, the intestines of pollinators, digestive tract of poultry, raw and fermented mammalian milk, fermented milk products, and wastewater (Biavati & Mattarelli, 2012). Generally, bifidobacteria are considered to be nonpathogenic, probiotic microorganisms playing an irreplaceable role in antipathogenic activities, the stimulation/maturation of the immune system, development of intestinal microbiota, and enzyme activities associated with the digestion of indigestible food components (O'Callaghan & van Sinderen, 2016). Nevertheless, some bifidobacteria may participate in the formation of dental caries (Valdez et al., 2016) and urinary tract infection (Barberis et al., 2012). There are several case reports of bifidobacterial bacteremia in preterm infants and immunocompromised individuals (Avcin, Pokorn, Kitanovski, Premru, & Jazbec, 2015; Weber et al., 2015).

Representatives of the scardovial genera differ from bifidobacteria based on genotypic, phenotypic characteristics, and habitat (Killer et al., 2013a). In most cases, they are adapted to an oxygen‐containing environment. They were found to be present in the human and animal oral cavity, human clinical samples, urinary tract, porcine cecum, and intestinal tract of wild pigs (Downes et al., 2011; Huys et al., 2007; Killer, Havlik, Bunesova, Vlkova, & Benada, 2014; Simpson, Ross, Fitzgerald, & Stanton, 2004). In contrast to bifidobacteria, scardovia inhabiting the human body are presented as opportunistic pathogens causing oral diseases and urinary tract infection (Mahlen & Clarridge, 2009; Tanner et al., 2011).

The classification and phylogenetic studies of bifidobacteria were based on the determination of carbohydrate fermentation patterns, enzyme activities, morphological and physiological characteristics, peptidoglycan type, DNA G + C content, DNA‐DNA relatedness, and 16S rRNA gene sequences by the end of the last century (Dong, Xin, Jian, Liu, & Ling, 2000; Scardovi, Trovatelli, Biavati, & Zani, 1979). Even though all of the above classification techniques, and especially the latter two, are still in use for the differentiation of taxonomic units and primary evaluation of phylogeny (Mattarelli et al., 2014), they have been reported to have several shortcomings. Biochemical and physiological characteristics can be similar among the strains of separated species and, on the other hand, vary among strains of the same species. The results of DNA G + C determination using traditional methods may differ from laboratory to laboratory, depending on the technique used (Fournier, Suhre, Fournous, & Raoult, 2006). DNA‐DNA heteroduplexes are formed between strands having at least 80% sequence complementarity. For this reason, DNA reassociation values may not replace the comparison of whole‐genome sequences and gene content differences (Lugli et al., 2014). Phylogeny, classification, and ecological studies on bifidobacteria based on the 16S rRNA gene can be misleading due to the presence of different copies in genomes (Satokari, Vaughan, Akkermans, Saarela, & de Vos, 2001; Větrovský & Baldrian, 2013; Wu, Jospin, & Eisen, 2013). Moreover, there are examples of bifidobacterial species sharing very similar 16S rRNA gene sequences (Delcenserie et al., 2007; Lugli et al., 2014). The application of variable regions within housekeeping genes belonging to COGs (Clusters of Orthologous Genes/Groups of proteins) family in the phylogeny of bifidobacteria is considered to be an alternative to 16S rRNA‐derived phylogeny (Jian, Zhu, & Dong, 2001; Killer, Sedláček, Rada, Havlík, & Kopečný, 2013b; Ventura et al., 2006).

A phylogenetic/identification marker applicable to almost the entire *Bifidobacteriaceae* family has not been devised yet. This led us to find a candidate gene in the genomes of representatives of the family with more robust discriminatory power among taxonomic units and comparable or better phylogenetic features than the 16S rRNA gene. The gene encoding the CTP (cytidine triphosphate) synthase (catalyzing the ATP‐dependent amination of UTP to CTP with either l‐glutamine or ammonia as the source of nitrogen), which plays an irreplaceable role in the synthesis of RNA in the process of transcription seems to be an appropriate candidate. It is ubiquitous in bacteria, homologous, exists in a single copy in the genome, is subject to stabilizing selection, stable with respect to rapid genetic modification and able to produce a robust phylogenetic tree that reflects the evolution of the species as much as possible (Glaeser & Kämpfer, 2015; Wu et al., 2013).

Below, we discuss the applicability of the variable region within the *pyrG* gene encoding the CTP synthase in the differentiation of taxonomic units and phylogenetic assessment of the *Bifidobacteriaceae* family compared with the 16S rRNA gene.

## 2 MATERIALS AND METHODS

### 2.1 Strains, culture conditions, and DNA extraction

Forty‐three type strains of the *Bifidobacteriaceae* family and seven wild strains of bifidobacteria (Table 1) of human and animal origin were used in the study. Type strains were purchased from the DSMZ (Leibniz Institute DSMZ – German Collection of Microorganisms and Cell Cultures). Sequences of the strain *B. thermophilum* JCM 1207^T^ were also included in the study because results of our recently accepted study (Pechar, Killer, Mekadim, Geigerová, & Rada, 2017a) confirmed a significant genetic difference between the strain and *B. thermophilum* DSM 20210^T^. Thus, we wanted to find out whether these two strains, both referred to as type, also differ based on the sequences of the given gene. All strains (except of *B. thermophilum* JCM 1207^T^) were routinely cultivated in anaerobic TPY broth (Scardovi, 1986) at 37°C for 24 h. Genomic DNA was extracted for PCR purposes from 1 ml of vital cultures using a DNeasy Blood & Tissue kit (Qiagen) following the manufacturer′s instructions for gram‐positive bacteria.

**Table 1:** Strains used in this study with NCBI numbers of 16S rRNA and *pyrG* genes

Strain	16S rRNA gene NCBI numbers	*pyrG* gene NCBI numbers
*Aeriscardovia aeriphila* DSM 22365^T^	NR042759	KT351222
*Alloscardovia omnicolens* DSM 21503^T^	AM419460	KT351223
*Alloscardovia criceti* DSM 17774^T^	NR041347	KT351224
*Parascardovia denticolens* DSM 10105^T^	D89331	KT351225
*Pseudoscardovia suis* DSM 24744^T^	NR118047	KT351226
*Pseudoscardovia radai* DSM 24742^T^	HQ842704	KT351227
*Scardovia inopinata* DSM 10107^T^	NR112093	KT351228
*Bifidobacterium adolescentis* DSM 20083^T^	NC008618	KY365559
*B. adolescentis* VB‐ES42	KY705017	KY746715
*B. angulatum* DSM 20098^T^	D86182	KT351229
*B. animalis* subsp. *animalis* DSM 20104^T^	NZJGYM01000004	KT351230
*B. animalis* subsp. *lactis* DSM 10140^T^	CP001606	NC012815
*B. asteroides* DSM 20089^T^	LC071851	KT351231
*B. biavatii* DSM 23969^T^	NZJGYN01000007	KT351232
*B. bifidum* DSM 20456^T^	NZJGYO01000002	KY365560
*B. bifidum* VB‐MA1	KY705018	KY746716
*B. bohemicum* DSM 22767^T^	NZJGYP01000004	KT351233
*B. bombi* DSM 19703^T^	NR104872	KT351234
*B. boum* DSM 20432^T^	NZJGYQ01000004	KT351235
*B. breve* DSM 20213^T^	AB006658	KT351236
*B. breve* VB‐TA1	KY705019	KY746717
*B. callitrichos* DSM 23973^T^	NZJGYS01000032	KT351237
*B. catenulatum* VB‐J50	KY705020	KY746718
*B. coryneforme* DSM 20216^T^	NZCP007287	KT351238
*B. crudilactis* LMG 23609^T^	NZJHAL01000001	KT351239
*B. dentium* DSM 20436^T^	AP012326	KT351240
*B. gallinarum* DSM 20670^T^	D86191	KT351241
*B. choerinum* DSM 20434^T^	NZJGYU01000002	KT351242
*B. indicum* DSM 20214^T^	NR043439	KT351243
*B. kashiwanohense* DSM 21854^T^	NR112779	KT351244
*B. longum* subsp. *infantis* DSM 20088^T^	AP010889	KT351245
*B. longum* subsp. *longum* DSM 20219^T^	AP010888	KT351246
*B. longum* subsp. *suis* DSM 20211^T^	NZJGZA01000002	KT351247
*B. longum* subsp. *suis* VB‐5/9	KY705021	KY746719
*B. merycicum* DSM 6492^T^	NZJGZC01000002	KT351248
*B. minimum* DSM 20102^T^	NZJGZD01000001	KT351249
*B. pseudocatenulatum* DSM 20438^T^	D86187	KT351250
*B. pseudocatenulatum* VB‐MA7	KY705022	KY746720
*B. pseudolongum* subsp. *globosum* DSM 20092^T^	D86194	KT351251
*B. pseudolongum* subsp. *pseudolongum* DSM 20099^T^	D86195	KT351252
*B. psychraerophilum* DSM 22366^T^	NZJGZI01000007	KT351253
*B. saguini* DSM 23967^T^	NZJGZN01000001	KT351254
*B. scardovii* DSM 13734^T^	NZJGZO01000008	KT351255
*B. stellenboschense* DSM 23968^T^	NZJGZP01000012	KT351256
*B. thermacidophilum* subsp. *porcinum* DSM 17755^T^	AY148470	KT351257
*B. thermacidophilum* subsp. *porcinum* VB‐T15	KY705023	KY746721
*B. thermacidophilum* subsp. *thermacidophilum* DSM 15837^T^	NZJDTO01000023	KT351258
*B. thermophilum* DSM 20210^T^	NZJDUB01000036	KT351259
*B. thermophilum* JCM 1207^T^	NZJGZV01000001	NZJGZV01000002
*B. tsurumiense* DSM 17777^T^	NZJGZU01000004	KT351260

^T^, type strain.

### 2.2 Isolation and identification of wild strains of bifidobacteria

Seven strains of bifidobacteria originating from the feces of infants and calves (Table S1) were isolated from colonies grown in modified TPY agar (Rada & Petr, 2000) under conditions previously reported (Killer et al., 2013a). They were then identified based on 16S rRNA gene sequences that were obtained after amplification using the 27f ‐ 1541R primer pair (Dong et al., 2000). The sequencing of almost complete 16S rRNA genes and the others in the study was performed by the company SEQme (CZ) on the basis of both primers. Complete sequences derived from forward and reverse primers were then stacked in the program Geneious v7.1.7 (Biomatters Ltd.) and stored in the GenBank database of the NCBI (National Centre for Biotechnology Information) through the application Banklt. The EzBioCloud database (Yoon et al., 2017) was used to find the closest related taxa.

### 2.3 Designing primers flanking the variable region of the *pyrG* gene

To design primers for the amplification and sequencing of the *pyrG* gene region of the family *Bifidobacteriaceae*, the corresponding sequences derived from the complete genome sequences belonging to 15 representatives of the family were applied (Table S2). The sequences were aligned using the Alignment tool in Geneious, which also automatically determined the direction to acquire the consensus sequence. This was used to design primers defining a variable fragment of the particular gene using the application Primer3 in the same software. In order to increase the specificity and functionality of the primers, PCR parameters such as the range of primer size, melting temperature (Tm), % GC, and then maximum Tm difference, maximum dimer Tm, maximum 3′ stability and GC clamp were properly set (Untergasser et al., 2012). The following primers covering a 798‐bp fragment (corresponding to the position 894819–895616 within the complete genome of *Bifidobacterium adolescentis* ATCC 15703^T^; NCBI accession number AP009256) were proposed: BifcPyrGF (5′‐ BCAYATCACCAACGARATYAA‐3′) and BifcPyrGR (5′‐ AYTCRATGACCATGGACTGCA‐3′). To be applicable to the widest range of different species of the *Bifidobacteriaceae* family, primers were manually corrected at some positions.

### 2.4 PCR amplification, sequencing, and deposition in the GenBank database

The *pyrG* gene fragment in representatives of the family *Bifidobacteriaceae* was amplified in a 25 μl PCR mixture composed of 1× PPP Master Mix (Top‐Bio, CZ; 75 mmol/L Tris‐HCl, pH 8.8, 20 mmol/L (NH_4_)_2_SO_4_, 0.01% Tween 20, 2.5 mmol/L MgCl_2_, 200 μmol/L of each deoxynucleoside triphosphate, 1.25 U *Taq* ‐ purple DNA polymerase), 0.2 μmol/L of each primer, and 10–100 ng of template DNA. The optimal PCR amplification program determined using the TProfessional Gradient 96 thermocycler (Biometra, Germany) was as follows: an initial denaturation at 95°C for 6 min, 32 cycles of denaturation at 95°C for 50 s, annealing at 53°C for 55 s, and extension at 72°C for 1 min; the amplification finished with a final extension step at 72°C for 7 min.

PCR products were analyzed in the presence of DNA marker 200–1,500 (Top‐Bio, CZ) on 1.5% agarose gel containing ethidium bromide (10 mg/ml) to ensure that a fragment of the correct size had been amplified. Electrophoresis was performed at 115 V for 40 min. Checked amplicons were purified with a PCR purification kit (Qiagen) and sequenced with the corresponding primers by SEQme (CZ). Final sequences derived from forward and reverse primers were constructed in Geneious. Sequencing of the particular gene fragment was verified using the nucleotide BLAST (Basic Local Alignment Search Tool) application that is part of the NCBI database containing all available genome and gene sequences. The assembled sequences were deposited after checking and editing in the GenBank database with the application Banklt.

### 2.5 Determination of gene characteristics

The alignment of sequences was carried out using the ClustalW algorithm in the program BioEdit v7.2.6 (http://www.mbio.ncsu.edu/BioEdit/page2.html). The partial *pyrG* and also 16S rRNA gene sequences were applied. The latter were retrieved for type strains from complete genomes (the GenBank/EMBL/DDBJ accession numbers are shown in Table 1). The 16S rRNA gene, generally considered to be a standard identification and phylogenetic marker in prokaryotes, was applied as a comparative baseline to verify whether the *pyrG* gene represents an appropriate molecular marker for the *Bifidobacteriaceae* family. The resulting alignment was subjected to the removal of hypervariable positions using the Gblocks algorithm with the default setting (Castresana, 2000). The percentages of mean pairwise identities, pairwise distances, identical sites, and CG contents were computed in Geneious v7.1.7 (Biomatters Ltd.). The other gene parameters, consisting of the mean number of nucleotide differences, *P*‐ distance (number of base substitution per site), *K*‐ distance, number of conserved and variable sites, number of parsimonious‐informative, and singleton variable sites were calculated in the software package MEGA v5.05 (Tamura et al., 2011). The latter four parameters were applied to the *pyrG* gene and amino‐acid sequences that were generated using the toggle translation tool in the program BioEdit. The program DnaSP v5 (Librado & Rozas, 2009) was used for calculation the number of synonymous sites (substitution in the coding region that causes no amino acid changes), nonsynonymous sites (substitution in the coding region that causes amino acid changes), nonsynonymous changes per nonsynonymous site (d*N*), synonymous changes per synonymous site (d*S*), and the ratio of d*N*/d*S* in the *pyrG* region.

### 2.6 Phylogenetic analyses

The best‐fit ML (Maximum Likelihood) evolutionary model for 16S rRNA and *pyrG* gene (and amino‐acid) datasets was determined in MEGA v5.05. The resulting models were then used for the phylogenetic tree in the same software. Bootstrap values were calculated after 1,000 replicates to estimate the reliability of the phylogenetic groups and clusters.

To test the incongruence length difference (ILD) between the 16S rRNA and *pyrG* tree topologies (*p *=* *.05), the BioNJ congruence analysis implemented in the software MLSTest (Tomasini, Lauthier, Llewellyn, & Diosque, 2013) was performed. The Templeton test, as a part of the NJ localized incongruence difference analysis in the same software, was also carried out. It analyses incongruence node by node in the topology of concatenated loci. A *p* value lower than .05 in a branch suggests that at least one locus is incongruent with the tested node.

### 2.7 Testing of recombination

Potential recombination events in the partial *pyrG* gene sequences of examined *Bifidobacteriaceae* strains were scanned using the automated RDP analysis (Martin & Rybicki, 2000) in the program RDP4 v4.67 (Martin et al., 2010).

## 3 RESULTS

### 3.1 Comparison of 16S rRNA and *pyrG* gene characteristics and statistics

The *pyrG* (length of 798 pb) gene fragment was amplified and sequenced in *Bifidobacteriaceae* strains under the conditions described above. The assigned GenBank/EMBL/DDBJ accession numbers of the gene fragment and 16S rRNA gene sequences are listed in Table 1. Basic gene parameters calculated in bifidobacteria, scardovia, and the whole *Bifidobacteriaceae* family are shown in Table 2. The stretch of *pyrG* gene fragment examined did not contain any indels in bifidobacterial species. Only five insertions and deletions in a short region were observed in *Parascardovia denticolens*,* Scardovia inopinata,* and *Alloscardovia* species, as illustrated in Figure S1. Although the 16S rRNA gene alignments covered a much longer segment, the shorter *pyrG* gene region exhibited a much higher sequence variability among representatives of the investigated family. This is documented by lower percentages of mean pairwise identities, identical sites and, on the other hand, higher values of mean number of nucleotide differences, *P*‐and *K*‐distances. As expected, the highest sequence variability was found among the scardovial species (Table 2). A lower content of CG was calculated in scardovia for both genes.

**Table 2:** Comparison of basic gene and phylogenetic parameters between the 16S rRNA and *pyrG* sequences of the *Bifidobacteriaceae* strains examined

	Parameter	Bifidobacteria	Scardovia	f. *Bifidobacteriaceae*
16S rRNA	Length of gene fragment (nt)	1415	1337	1269
	Mean sequence similarities (pairwise identity) (%)	95.9	92.4	94.8
	Identical sites (%)	84.7	84.0	78.7
	Cytosine + Guanine (%)	59.7	59.3	59.9
	Mean number of nucleotide differences	56.65	100.24	62.50
	*P*‐distance (number of base substitution per site)	0.040	0.075	0.050
	*K*‐distance	0.042	0.080	0.052
	*Phylogenetic parameters*			
	AIC best fit ML model			TN93 + G + I
	Percentage of bootstrap values >50%			57.4
	Percentage of bootstrap values >70%			44.7
*pyrG*	Length of gene fragment (nt)	798	795	795
	Mean sequence similarities (pairwise identity) (%)	84.4	73.3	81.7
	Identical sites (%)	52.5	49.8	43.3
	Cytosine + Guanine (%)	62.7	57.9	62.0
	Mean number of nucleotide differences	124.42	211.95	145.68
	*P*‐distance (number of base substitution per site)	0.156	0.267	0.183
	*K*‐distance	0.178	0.337	0.216
	*Phylogenetic parameters*			
	AIC best fit ML model (for amino acid phylogenetic tree)			TN93 + G + I (WAG + G + I)
	Percentage of bootstrap values >50% (amino acid phylogenetic tree)			64.6 (59.5)
	Percentage of bootstrap values >70% (amino acid phylogenetic tree)			52.1 (38.1)

Percentages of *pyrG* gene pairwise distances among all strains tested are presented in Table S3. In scardovial species, the lowest and highest values were computed between *Aeriscardovia aeriphila*‐*Alloscardovia criceti* (66.29%) and *Pseudoscardovia suis*‐*Pseudoscardovia radai* (89.69%), respectively. In terms of bifidobacterial species, the highest and lowest distances were determined between *B. indicum‐B. saguini* (74.72%) and *B. catenulatum* VB‐J50‐*B. kashiwanohense*,* B. kashiwanohense*‐*B. pseudocatenulatum* (both pairs 98.11%), respectively.

Basic *pyrG* gene statistics revealed a higher number of variable and nonsynonymous sites than conserved and synonymous sites in the whole family and in scardovia. The opposite was found for amino acid sequences (Table 3). The ratio of d*N*/d*S* < 1 (0.12–0.23 in bifidobacteria, scardovia and the whole *Bifidobacteriaceae* family) indicates that most of the sequence variability can be explained by purifying (stabilizing) selection and slow evolution.

**Table 3:** DNA polymorphism in the *pyrG* gene (and derived amino acids) among *Bifidobacteriaceae* strains tested. Length of sequences presented in Table 2 was applied for computation

	C (IS)	VS	PI	SVS	SS	NSS	d*N*	d*S*	d*N*/d*S*
*Bifidobacteriaceae* (265 amino acids)	344 (148)	451 (117)	394 (90)	57 (27)	194.44	600.56	0.08041	0.50128	0.16
Bifidobacteria (266 aa)	419 (189)	379 (77)	333 (62)	46 (15)	195.24	602.76	0.05586	0.46492	0.12
Scardovia (265 aa)	396 (161)	399 (104)	260 (55)	139 (49)	191.52	603.48	0.14643	0.64571	0.23

C, number of conserved (invariable sites); VS, number of variable sites; PI, number of parsimonious‐informative sites; SVS, number of singleton variable sites; SS, number of synonymous sites; NSS, number of nonsynonymous sites; d*N*, number of nonsynonymous changes per nonsynonymous site; d*S*, number of synonymous changes per synonymous site.

### 3.2 Classification of wild strains of bifidobacteria

The classification of seven wild strains of bifidobacteria through 16S rRNA and *pyrG* gene comparative analysis is documented in Table S1. The strains originating from infant feces were classified as *B. adolescentis*,* B. bifidum*,* B. breve*,* B. catenulatum,* and *B. pseudocatenulatum*, whereas those from the feces of calves as *B. longum* subsp. *suis* and *B. thermacidophilum* subsp. *porcinum*. In all these strains (except for *B. catenulatum*), better differentiation from type strains was found using the *pyrG* gene than with the 16S rRNA gene sequences.

### 3.3 Phylogenetic analyses

**Figure 1:**
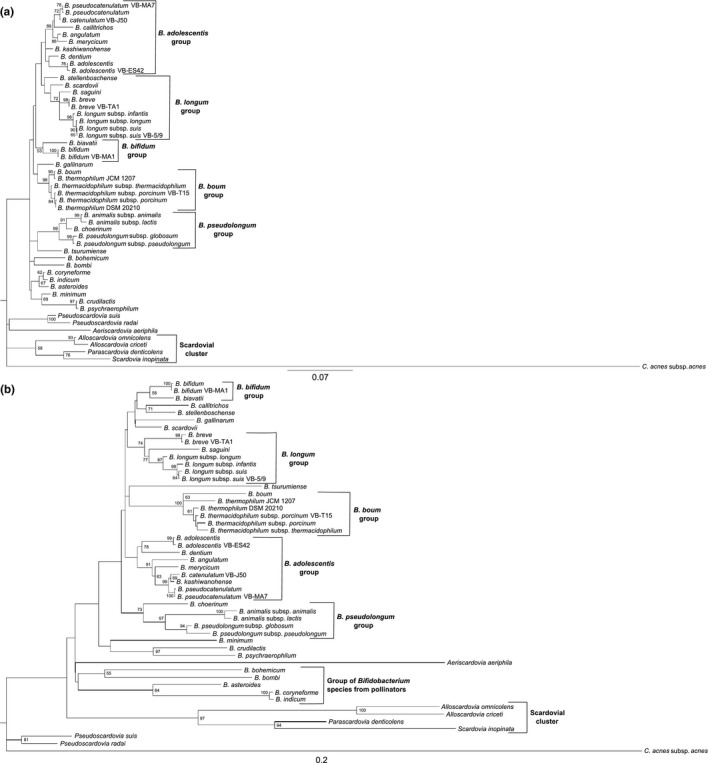
Phylogenetic relationships among representatives of the family *Bifidobacteriaceae* based on trees reconstructed through: (a) 16S rRNA gene sequences (1,269 nt) and (b) partial *pyrG* gene sequences (795 nt). Maximum‐likelihood statistical method and AIC best fit ML model (Table 2) implemented in MEGA v5.05 software were applied. Phylogeny was improved by bootstrapping (1,000 datasets). Bootstrap percentages (>50) are given at nodes. Trees were rooted by *Cutibacterium acnes* subsp. *acnes* ATCC 6919^T^ (for 16S rRNA‐derived phylogeny, GenBank accession number AB042288), DSM 1897^T^ (for *pyrG*‐derived phylogeny, NZAWZZ01000003). Bars refer to substitutions per nucleotide position

The phylogeny of the family *Bifidobacteriaceae* reconstructed based on 16S rRNA and *pyrG* gene sequences is presented in Figure 1. The maximum‐likelihood algorithm and the best‐fit ML evolutionary model (Table 2) were applied. The 16S rRNA‐derived phylogenetic tree, created primarily by sequences found in complete genomes to make the topology as accurate as possible, includes a separated scardovial cluster and *B. adolescentis*,* B. bifidum*,* B. boum*,* B. longum,* and *B. pseudolongum* phylogenetic groups (Figure 1a). The same phylogenetic groups, plus a cluster including species of bifidobacteria isolated from important pollinators (honeybees and bumblebees) are visible in the *pyrG* phylogenetic tree (Figure 1b). The higher confidence, more accurate topology and robustness of *pyrG*‐based phylogeny are documented by higher percentages of bootstrap values >50 and >70 (Table 2). The much higher discriminatory power among *Bifidobacteriaceae* species in the *pyrG*‐derived phylogeny is shown by the length of branches and the scale referring to substitutions per nucleotide position.

A comparative analysis revealing the degree of differentiation of bifidobacterial subspecies based on 16S rRNA and *pyrG* gene sequences was also performed. A higher resolution was determined in the *pyrG* gene sequences, as shown in Table S4.

The phylogeny based on *pyrG* gene‐derived amino‐acids (265 aa) sequences revealed a separated cluster of scardovia, then the *B. boum* and *B. pseudolongum* phylogenetic groups and a cluster including *Bifidobacterium* species from pollinators (Figure S2). The phylogenetic groups of *B. adolescentis*,* B. bifidum,* and *B. longum* are placed together in the supercluster.

As expected, the ILD testing between the 16S rRNA and *pyrG* trees (*p *=* *.01) revealed a length incongruence, and the topology of the concatenated loci should be considered carefully. Understandably, coding and noncoding genes should not be combined. Nevertheless, we also performed the NJ localized incongruence difference analysis based on the Templeton test just to find out if the topologies of 16S rRNA and *pyrG*‐based phylogenetic trees are in (in)congruence. The phylogenetic tree showing node by node statistical values revealing whether at least one gene is (in)congruent with the tested node is presented in Figure S3. All phylogenetic branches reconstructed based on the concatenation of 16S rRNA and *pyrG* gene sequences are congruent, as documented by *p* values >.05.

### 3.4 Assesment of recombination events

Recombination events in the *pyrG* gene sequences of the examined *Bifidobacteriaceae* strains were not observed using the automated RDP analysis.

## 4 DISCUSSION

The *pyrG* gene encoding the CTP synthase is ubiquitous in all bacteria, present in a single copy in genomes, long enough to contain sufficient information, nondiscriminatory to horizontal gene transfer or recombination and contains at least two highly conserved regions allowing the proposed primers to delineate the variable region (Adékambi et al., 2011; Roux, Enault, Bronner, & Debroas, 2011; Wu et al., 2013). Therefore, this gene encoding a protein implicated in nucleotide metabolism seems to be an appropriate candidate for a molecular marker usable in the identification, classification, typing, and phylogeny of bacteria. It was applied in various taxonomic groups of bacteria, for example, in the genus *Borrelia*,* Edwardsiella*, order Xanthomodales, various *Lactobacillus* species and for assessing microbial diversity in ecosystems (Abayneh, Colquhoun, & Sørum, 2012; Diancourt et al., 2007; Naushad & Gupta, 2013; Roux et al., 2011; Rudenko, Golovchenko, Belfiore, Grubhoffer, & Oliver, 2014; Sarmiento‐Rubiano et al., 2010).

Average nucleotide identity of *pyrG* gene sequences (Table 2) calculated in bifidobacteria (84.4%) and the whole family (81.7%) is to some extent similar to that calculated among species of bifidobacteria based on *clpC* (81.35%), *hsp60* (85.0%), and *rpoC* (88.25%) gene sequences (Jian et al., 2001; Ventura et al., 2006). Nonetheless, it is important to note that shorter gene segments (540–690 nt) and a lower number of *Bifidobacterium* species were considered. A lower content of CG determined in scardovia for both genes is consistent with previous findings based on the values of this parameter in genomic DNA (Killer et al., 2010, 2013a). The average GC composition of the *pyrG* gene calculated in all strains tested (62.0%) is typical for bacteria belonging to the *Actinobacteria* phylum (Delétoile et al., 2010).

The calculated ratio of d*N*/d*S* < 1 on the basis of *pyrG* gene sequences among *Bifidobacteriaceae* strains tested (Table 3) suggesting stabilizing selection and slow evolution together with no detection of recombination events allow the particular gene to be considered a suitable molecular marker (Glaeser & Kämpfer, 2015; Nuñez et al., 2014). Genes that are not prone to horizontal transmission and recombination meet one of the basic requirements for their use as identification and phylogenetic markers (Adékambi et al., 2011; Roux et al., 2011). It is necessary to point out that mainly type strains (with the exceptions of seven strains) were employed and thus intraspecies recombinations may not be ruled out.

Individual phylogenetic groups are better defined based on *pyrG* gene sequences in the contrast to the 16S rRNA‐derived phylogeny and the topology of the phylogenetic tree more accurately reflects those observed in phylogenetic studies of the genus *Bifidobacterium* and the *Bifidobacteriaceae* family on the basis of whole‐genomic assays (Lugli et al., 2014; Milani et al., 2014, 2015; Sun et al., 2015; Zhang, Gao, Adeolu, Khadka, & Gupta, 2016). Within these studies, the *B. pullorum* phylogenetic group was also mentioned. This group is missing in our study, because some species classified into the phylogenetic group (*B. pullorum* and *B. saeculare*) were not included in this study. Some discrepancies were found in the *B. longum* and *B. bifidum* phylogenetic groups in the *pyrG*‐based phylogeny compared with whole‐genomic phylogenies. Species *B. angulatum* and *B. merycicum* belong to the *B. longum* phylogenetic group, whereas these species are positioned in the *B. adolescentis* group based on the *pyrG* phylogenetic study (Figure 1b). The species *B. callitrichos* and *B. stellenboschense* clustered with the *B. bifidum* group near the *B. longum* group. However, these species are classified into the *B. longum* group using the whole‐genomic analyses (Lugli et al., 2014; Zhang et al., 2016). As illustrated in both trees (Figure 1a,b), strains *B. thermophilum* JCM 1207^T^ and *B. thermophilum* DSM 20210^T^ differ from each other. Strain *B. thermophilum* JCM 1207^T^ seems to be more related to *B. boum* type strain. Besides, Lugli et al. (2014) came to a similar conclusion. We therefore believe that the strain *B. thermophilum* DSM 20210^T^ should be considered a real‐type strain of a given species.

Classification and phylogenetic position of wild strains of bifidobacteria using *pyrG* gene sequences correlated with those found on the basis of 16S rRNA gene sequences (Table S1, Figure 1a,b). This allows to consider the *pyrG* gene as an appropriate, easily accessible identification tool for isolates of bifidobacteria of different origin.

All phylogenetic branches and nodes reconstructed based on the concatenation of 16S rRNA and *pyrG* gene sequences using the Templeton test implemented in NJ localized incongruence difference analysis are congruent (Figure S3). This indicates that the *pyrG* gene can be used as an alternative phylogenetic marker of the 16S rRNA gene in the *Bifidobacteriaceae* family. Remarkably, the robustness and topology of the tree encompassing particular taxonomic groups correspond to a large extent to the phylogenomic analyses of the family (Lugli et al., 2017), although 16S rRNA and a coding housekeeping gene‐phylogeny should not usually be applied together.

Although the application of a single gene fragment for classification and phylogenetic purposes in particular taxonomic groups of bacteria may in many ways be confusing and inaccurate, the results presented above provide evidence that the *pyrG* gene may be used in the family *Bifidobacteriaceae* as an alternative classification and phylogenetic marker to the 16S rRNA gene. In contrast to the phylogenetic marker used as the ‘gold standard’ in the phylogeny of prokaryotes, the higher degree of sequence divergence of the *pyrG* gene at the (sub)species level (Table S4) is superior for identification purposes. The gene is expected to be applicable for the classification of new *Bifidobacteriaceae* taxa and MLSA (MultiLocus Sequence Analysis). In the future, the application of larger numbers of strains of a given species should confirm or refute the integration of this gene into MLST (Multilocus sequence typing) analyses.

## CONFLICT OF INTEREST

The authors declare that they have no competing interests.
