# Supplementary material for: Gene encoding the CTP synthetase as an appropriate molecular tool for identification and phylogenetic study of the family Bifidobacteriaceae
Source: Microbiologyopen. 2018 Jan 22;7(4):e00579. doi: 10.1002/mbo3.579 (PMC6079163; doi:10.1002/mbo3.579)
Supplement: Supplementary file 1 [file MBO3-7-e00579-s001.pdf]

Supplementary Materials for:

Gene encoding the CTP synthetase as an appropriate molecular tool for identification and phylogenetic study of the family *Bifidobacteriaceae*

Jiří Killer, Chahrazed Mekadim, Radko Pechar, Věra Bunešová, Jakub Mrázek, Eva Vlková

Fig. S1 An image created in the BioEdit v7.2.6 program after ClustalW alignment showing a short fragment of *pyrG* gene in the family *Bifidobacteriaceae* containing insertions and deletions in scardovial species.

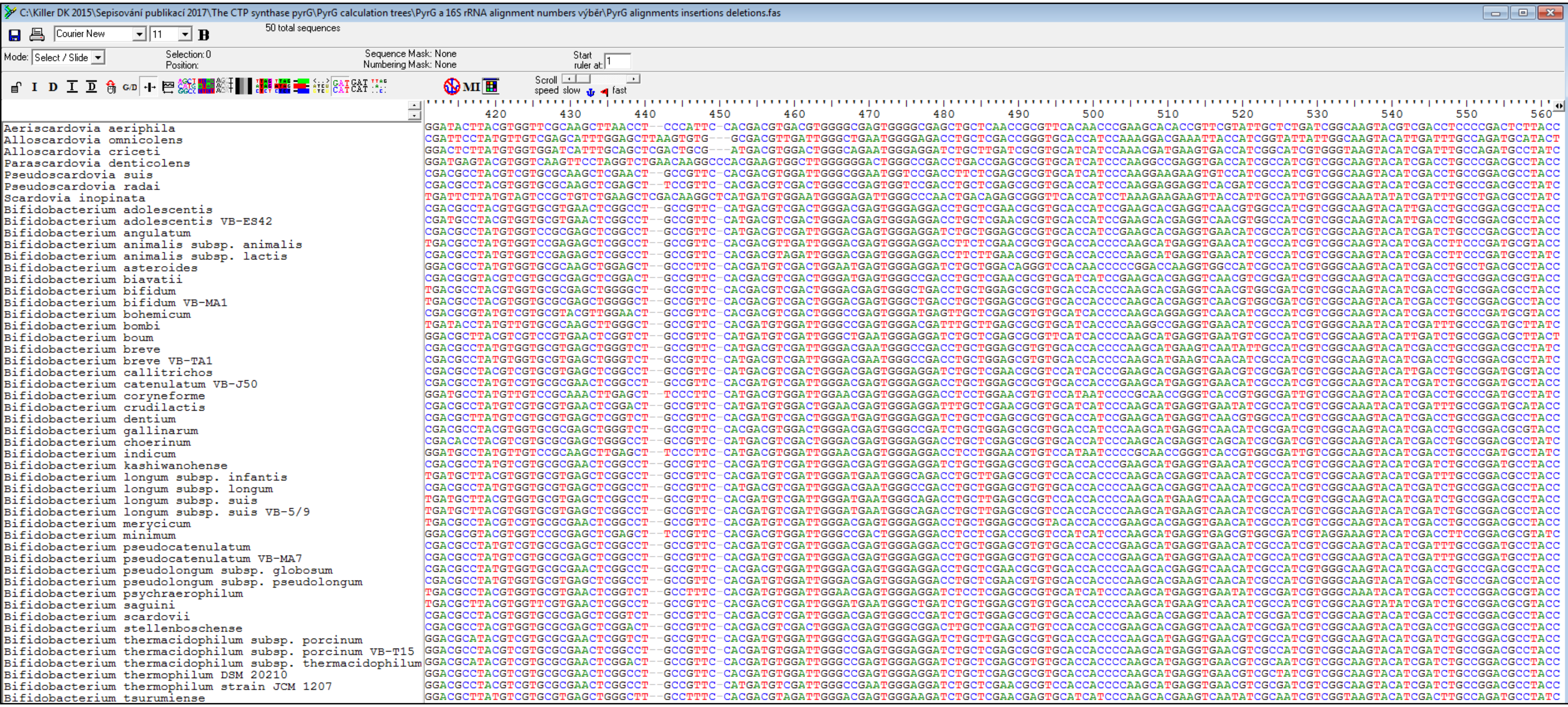

Fig. S2 Phylogenetic tree of the family *Bifidobacteriaceae* reconstructed using the *pyrG* gene-derived amino-acids (265 aa). Maximum-likelihood statistical method and AIC best fit ML model (**Table 2**) were used. Phylogeny was improved by bootstrapping (1000 replicates). Bootstrap percentages (> 50) are given at nodes. Trees were rooted by *Cutibacterium acnes* subsp. *acnes*. Bar, 0.06 substitutions per amino-acid position.

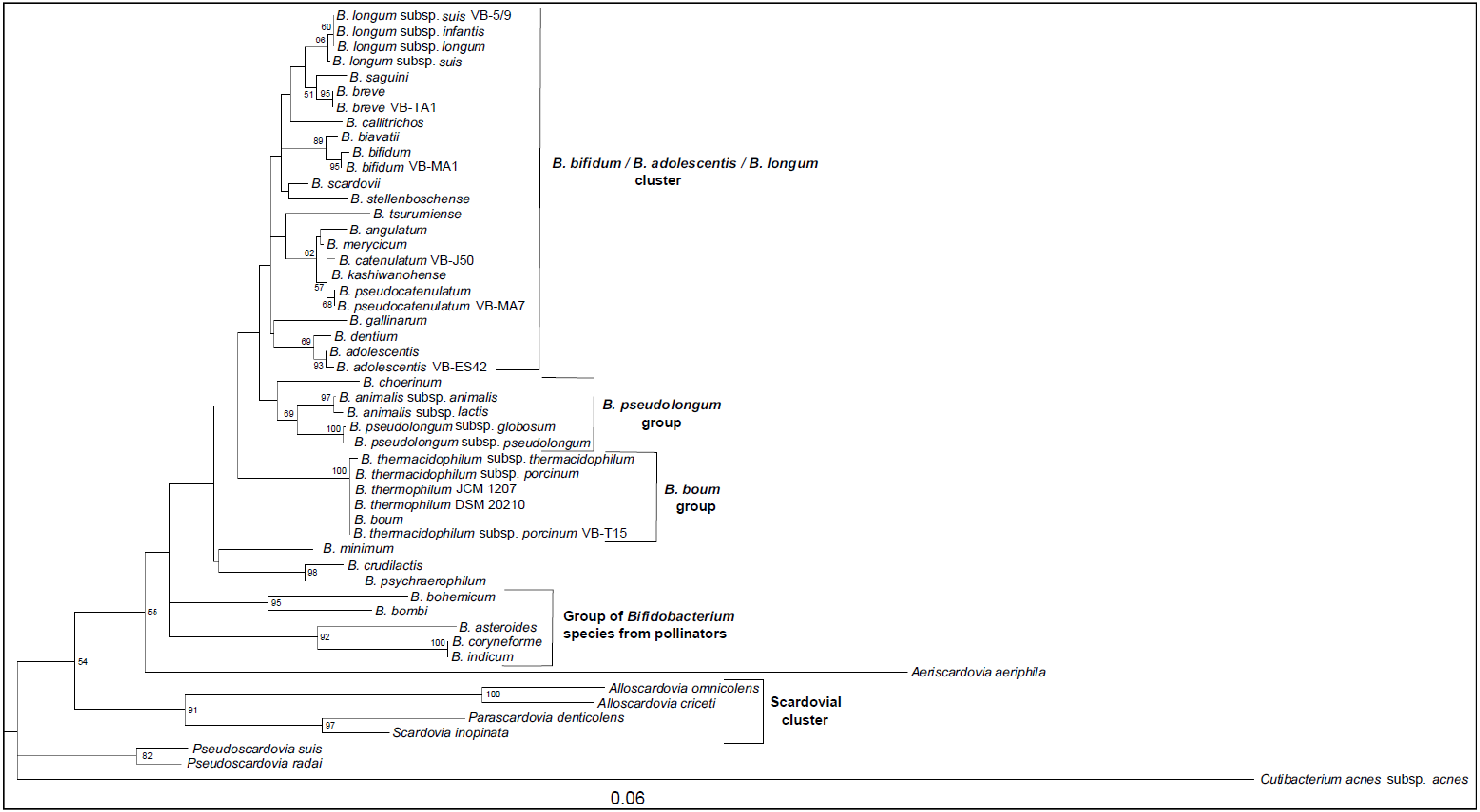

Fig. S3 Phylogeny of the family *Bifidobacteriaceae* using the NJ localized incongruence difference analysis based on Templeton test implemented in MLSTest software. The tree was reconstructed using the 16S rRNA (1269 nt) and *pyrG* (795 nt) gene sequences. Statistical values of probability (*p*) suggesting in a branch that at least one gene is incongruent ($p < 0.05$) with the tested node are given at nodes. Trees were rooted by *Cutibacterium acnes* subsp. *acnes*. Scale refers to difference in 40 nucleotides.

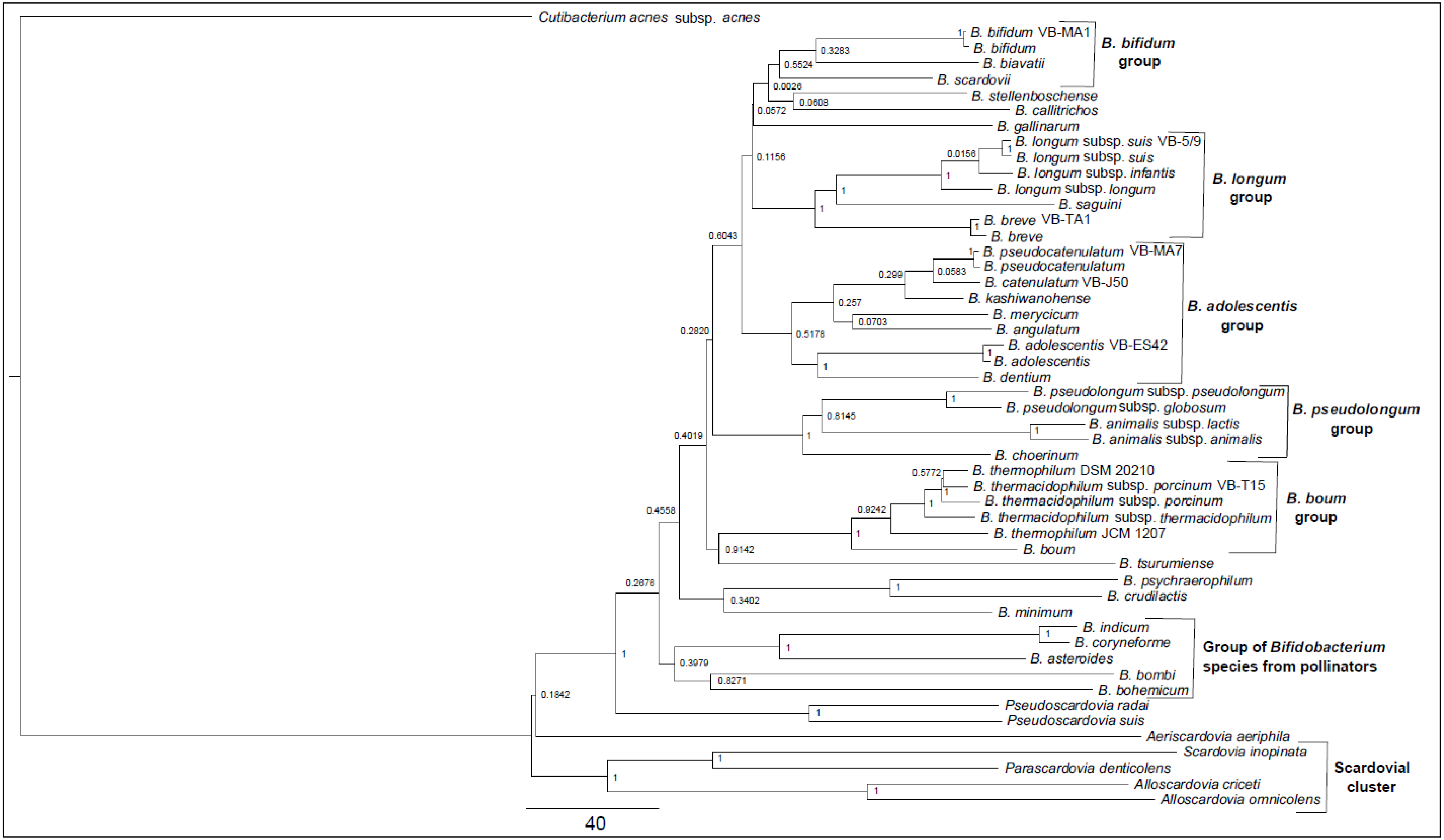

Table S1 Classification of wild bifidobacterial strains based on 16S rRNA (lenght 1485 nt) and pyrG (798 nt) gene identities (%).

Strain	The closest relative (NCBI accession number)	16S rRNA identity	*PyrG* identity	Origin
VB-ES42	*B. adolescentis* ATCC 15703T (AP009256)	99.79	99.37	infant feces
VB-MA1	*B. bifidum* ATCC 29521^T (KE993182)	99.86	99.75	
VB-TA1	*B. breve* DSM 20213^T (ACCG01000002)	100	99.12	
VB-J50	*B. catenulatum* DSM 16992^T (ABXY01000019)	99.79	99.88 (NCBI a. n. ABXY01000011)	
VB-MA7	*B. pseudocatenulatum* DSM 20438^T (ABXX02000002)	99.93	99.75	
VB-5/9	*B. longum* subsp. *suis* LMG 21814^T (JGZA01000002)	100	99.37	feces of calves
VB-T15	*B. thermacidophilum* subsp. *porcinum* LMG 21689^T (JGZS01000003)	99.72	98.12	

Table S2 Position of *pyrG* genes used for primer designing in genomes of representatives of the family *Bifidobacteriaceae*.

***Bifidobacteriaceae* strains**	***pyrG* (position)**
Alloscardovia omnicolens F0580	AWSI01000036 (1712-3373)
Parascardovia denticolens IPLA 20019	AKII01000017 (63525-65219)
Parascardovia denticolens DSM 10105^T	AEON01000001 (857736-859448)
Scardovia inopinata F0304	ADCX01000003 (290003-291685)
Scardovia wiggsiae F0424	AGZS01000001 (322654-324339)
Bifiddobacterium adolescentis ATCC 15703^T	NC008618 (894426-896087)
B. angulatum DSM 20098^T	ABYS02000004 (762319-763980)
B. animalis subsp. *lactis* DSM 10140^T	NC012815 (1058705-1060252)
B. asteroides PRL2011	CP003325 (1162482-1164146)
B. bifidum PRL2010	CP001840 (1001842-1003509)
B. breve UCC2003	CP000303 (1154882-1156546)
B. catenulatum DSM 16992^T	ABXY01000011 (552400-554061)
B. dentium Bd1	NC013714 (1164736-1166397)
B. longum subsp. *longum* JCM 1217^T	NC015067 (936123-937784)
B. thermophilum RBL67	CP004346 (1209421-1211088)

Table S3 Pairwise distances (%) among strains (numbers correspond to the strains according to **Table 1**) of the family *Bifidobacteriaceae* computed based on *pyrG* gene sequences (795 nt) using the Geneious version 7.1.7 software (Biomatters Ltd). Highlighted in red are the lowest and highest values among scardovial and bifidobacterial species.

	1	2	3	4	5	6	7	8	9	10	11	12	13	14	15	16	17	18	19	20	21	22	23	24	25	26	27	28	29	30	31	32	33	34	35	36	37	38	39	40	41	42	43	44	45	46	47	48	49
1	-																																																
2	66.92	-																																															
3	**66.29**	81.51	-																																														
4	69.56	72.33	72.96	-																																													
5	70.57	70.94	71.70	77.86	-																																												
6	72.58	69.18	70.94	78.37	**89.69**	-																																											
7	68.05	71.82	72.83	80.13	72.83	73.08	-																																										
8	73.33	69.94	70.82	76.48	82.26	83.02	71.57	-																																									
9	73.33	69.94	70.57	76.60	82.14	83.02	71.57	99.37	-																																								
10	71.45	70.69	71.95	76.35	79.75	80.63	71.07	89.69	89.56	-																																							
11	70.31	69.06	69.31	73.96	77.74	78.24	70.19	85.16	85.16	84.28	-																																						
12	69.94	68.68	69.56	73.96	78.49	79.25	70.44	85.54	85.54	85.03	96.98	-																																					
13	71.57	68.18	69.06	76.35	76.60	78.99	71.95	80.25	79.87	79.25	77.61	77.86	-																																				
14	72.45	70.06	69.56	76.98	84.28	84.78	69.81	90.69	90.57	88.18	83.40	83.52	80.00	-																																			
15	72.58	70.69	70.82	77.61	82.26	82.77	71.20	89.94	89.81	89.18	83.27	83.27	80.88	93.59	-																																		
16	72.83	70.44	70.82	77.48	82.52	82.89	71.32	90.19	90.06	89.18	83.40	83.40	81.01	93.59	99.75	-																																	
17	68.43	68.05	70.06	72.33	74.97	76.73	69.18	77.74	77.36	77.99	78.24	78.49	77.61	79.12	78.24	78.37	-																																
18	71.45	69.69	71.32	74.72	76.10	77.48	71.82	76.86	76.73	78.49	77.48	78.24	75.35	77.99	77.99	77.99	77.99	-																															
19	72.20	69.06	70.82	74.84	78.37	77.86	71.57	83.15	83.02	84.53	81.64	81.13	77.48	84.15	83.15	83.40	78.37	75.35	-																														
20	71.45	69.81	71.45	75.09	80.88	81.51	71.95	86.42	86.29	87.30	82.14	82.77	79.25	87.55	88.81	89.06	79.87	77.23	83.77	-																													
21	71.45	69.69	71.70	75.60	81.51	81.76	71.82	87.04	86.92	87.67	82.39	83.02	79.37	88.18	89.18	89.43	79.87	77.48	83.77	99.12	-																												
22	71.70	69.69	71.20	76.10	81.76	83.15	70.19	88.05	87.93	86.42	84.40	84.65	80.25	91.20	89.18	89.18	79.12	78.49	84.03	88.81	89.18	-																											
23	70.94	70.44	72.33	76.23	80.25	80.76	71.70	90.31	90.19	90.94	84.91	85.16	79.25	88.68	89.18	89.18	79.50	79.25	83.90	88.05	88.05	87.17	-																										
24	69.43	67.80	69.81	74.84	74.47	74.84	72.08	77.48	77.23	77.48	76.60	76.86	82.01	77.48	78.99	78.87	76.35	75.35	76.23	77.86	77.99	78.24	76.98	-																									
25	68.55	69.06	72.70	76.10	77.36	78.24	72.08	81.26	81.26	80.88	80.00	80.25	77.86	81.26	80.13	79.87	77.99	78.62	80.00	81.01	81.13	81.51	83.77	77.74	-																								
26	71.45	69.94	70.94	75.85	80.76	82.64	71.32	90.94	90.82	88.55	84.28	84.53	80.63	89.18	89.06	89.31	80.13	76.86	83.65	87.30	87.55	87.42	90.06	77.48	81.51	-																							
27	71.82	69.31	71.70	76.60	81.51	82.64	70.44	88.05	87.67	87.93	82.77	83.02	80.76	89.69	89.31	89.06	78.99	77.11	83.02	86.92	87.67	87.42	86.54	77.74	80.88	88.30	-																						
28	71.95	68.43	69.81	76.60	82.77	84.78	71.32	88.43	88.30	85.03	85.41	85.66	80.00	89.56	87.55	87.55	79.75	77.99	81.76	85.41	85.91	87.55	85.54	78.37	81.89	86.16	86.67	-																					
29	69.56	67.30	69.56	74.59	74.47	75.22	71.82	77.48	77.23	77.48	76.60	77.11	82.52	77.74	78.99	78.87	76.60	75.47	75.72	77.61	77.74	78.37	76.98	98.87	77.61	77.61	77.86	78.49	-																				
30	71.32	70.69	72.70	76.23	81.01	81.01	71.45	90.69	90.57	92.08	84.91	85.28	79.87	88.68	89.56	89.56	79.50	79.25	84.78	87.67	87.93	87.42	**98.11**	77.11	82.89	90.06	87.17	86.04	76.98	-																			
31	72.20	70.57	71.57	76.98	79.87	81.38	72.08	86.54	86.67	85.16	82.64	82.64	80.88	87.17	87.67	87.93	78.49	76.98	83.90	89.06	89.43	87.67	86.54	76.48	79.87	87.42	85.41	84.53	77.11	87.30	-																		
32	72.33	70.94	72.58	77.11	80.76	82.39	72.70	87.67	87.55	86.92	83.15	83.15	81.26	88.18	88.93	89.18	79.62	77.36	84.53	91.82	91.95	88.93	87.80	77.86	81.13	87.93	86.42	86.16	77.99	88.05	95.85	-																	
33	72.58	70.69	71.95	77.23	80.13	81.26	72.45	86.67	86.79	85.28	83.15	82.64	80.50	87.30	87.93	88.18	78.87	76.73	84.40	89.81	90.44	87.80	86.79	76.48	80.25	87.42	85.79	84.91	76.60	87.55	98.11	95.98	-																
34	72.58	70.82	71.95	77.36	80.25	81.38	72.58	86.54	86.67	85.16	83.40	82.89	80.76	87.17	87.80	88.05	78.87	76.23	84.28	89.81	90.19	87.67	86.79	76.60	80.25	87.42	85.66	84.78	76.73	87.30	98.74	96.10	99.37	-															
35	71.95	70.44	71.95	76.10	81.26	81.64	71.32	90.06	89.69	91.70	84.03	84.15	80.13	89.31	89.94	89.94	78.74	78.37	84.65	86.67	86.92	87.30	92.96	76.98	81.26	88.81	87.55	86.16	76.86	93.96	87.55	88.05	87.80	87.55	-														
36	70.57	68.43	71.20	75.85	79.37	81.26	70.69	83.15	83.15	82.77	81.76	82.52	78.62	84.78	83.65	83.77	75.72	77.48	79.62	81.51	81.51	83.27	83.27	76.86	80.50	83.40	82.26	84.91	77.36	82.89	81.76	82.52	81.89	82.01	83.27	-													
37	70.94	70.69	71.95	75.85	81.38	81.38	71.57	90.31	90.19	91.70	84.78	84.91	79.87	88.81	89.43	89.43	79.62	79.50	84.53	87.67	87.93	87.04	96.98	77.36	82.64	89.43	87.04	86.29	77.48	**98.11**	87.17	88.18	87.42	87.17	93.59	82.77	-												
38	70.94	70.69	71.95	75.98	81.38	81.38	71.57	90.31	90.19	91.70	85.03	85.16	79.87	88.81	89.43	89.43	79.87	79.50	84.53	87.93	87.93	87.04	97.23	77.36	82.64	89.69	87.04	86.29	77.48	**98.11**	87.42	88.43	87.42	87.42	93.59	82.89	99.75	-											
39	71.82	68.18	70.19	76.48	81.64	82.01	70.94	88.55	88.43	86.29	87.93	88.81	80.76	86.92	86.04	86.29	79.12	78.49	82.14	85.03	85.66	86.04	86.29	78.62	81.01	87.04	86.92	90.19	78.49	86.92	85.91	86.92	86.16	86.04	86.29	83.52	86.54	86.54	-										
40	70.82	67.55	69.81	74.47	80.3	80.13	71.07	86.79	86.67	85.03	87.17	88.18	80.25	84.91	84.78	85.03	78.49	77.36	82.14	84.78	85.41	84.65	84.40	77.99	80.38	86.04	85.28	88.05	77.74	84.91	84.53	85.79	85.16	84.91	84.65	82.01	85.66	85.66	95.72	-									
41	70.44	69.06	70.82	75.22	77.74	77.74	70.31	80.76	80.63	79.50	79.75	79.87	77.23	80.88	81.64	81.51	75.35	76.23	80.63	79.62	80.00	80.63	79.87	77.61	83.65	80.25	81.64	81.01	77.74	80.25	80.38	79.75	80.13	80.13	81.26	81.64	79.87	79.87	81.76	81.64	-								
42	70.82	70.19	72.70	75.98	79.37	80.00	72.83	85.41	85.28	86.29	81.26	81.51	78.37	85.41	86.29	86.54	77.862	74.34	84.65	87.67	88.05	86.29	86.29	75.09	79.75	86.04	85.91	83.65	**74.72**	86.42	88.93	90.06	89.94	89.94	85.91	81.13	85.79	85.79	84.53	83.65	79.12	-							
43	71.95	68.93	70.94	77.36	83.65	85.03	70.57	89.56	89.43	89.06	85.03	85.66	80.88	92.08	92.33	92.58	79.12	77.99	84.53	89.31	89.94	89.31	88.93	77.36	81.38	88.68	90.19	89.56	77.61	89.69	90.19	91.20	89.81	89.94	89.31	84.65	89.81	89.81	90.06	88.30	81.89	87.30	-						
44	71.20	68.81	69.69	76.60	82.52	84.28	70.69	88.30	88.18	88.05	83.65	84.53	81.13	92.33	90.57	90.57	80.38	78.87	83.02	88.05	88.55	91.57	87.42	78.62	81.01	86.79	89.06	88.93	78.99	87.67	87.42	88.43	87.30	87.17	88.55	83.40	88.30	88.30	88.18	87.30	81.26	85.41	91.95	-					
45	71.20	69.06	69.69	75.85	80.63	80.00	70.94	85.28	85.16	85.16	82.89	83.40	78.99	86.29	85.79	86.04	79.62	77.23	90.31	85.03	85.41	85.66	85.66	76.60	81.64	85.54	86.42	84.91	76.48	85.54	85.28	85.91	85.66	86.04	85.91	82.64	85.41	85.66	85.66	84.78	81.64	85.16	87.93	85.91	-				
46	71.82	69.43	70.06	76.23	80.88	80.50	70.82	86.16	86.04	86.16	83.02	83.27	79.12	86.79	86.42	86.67	79.50	76.86	90.06	85.41	85.79	86.16	86.54	76.86	81.76	85.91	87.04	85.41	76.60	86.42	85.66	86.54	86.04	86.42	86.42	82.77	86.29	86.54	86.16	84.65	81.38	85.41	88.18	86.42	98.11	-			
47	71.45	69.43	69.94	75.22	80.38	80.00	70.82	85.16	85.03	85.66	82.77	83.27	78.49	86.54	85.79	86.04	79.62	76.35	89.56	85.03	85.41	85.28	85.54	76.86	81.01	85.16	86.54	84.28	76.86	85.41	85.16	85.91	85.28	85.66	85.91	81.89	85.79	86.04	85.54	84.40	81.51	85.28	88.05	86.42	97.11	97.61	-		
48	71.82	69.81	70.19	76.35	81.01	80.38	71.07	86.04	85.91	86.42	83.27	83.52	78.99	86.67	86.54	86.79	79.50	76.73	89.94	85.41	85.79	86.42	86.16	77.23	81.51	86.04	86.79	85.28	76.73	86.54	85.41	86.29	85.79	86.16	86.54	82.52	86.42	86.67	85.91	84.78	82.01	85.66	88.05	86.67	97.61	98.49	97.23	-	
49	70.94	69.56	69.69	74.59	79.50	79.25	71.07	84.91	84.78	85.66	81.76	81.76	78.87	85.79	85.66	85.91	77.99	76.10	89.69	85.41	85.54	86.16	85.66	76.73	79.75	84.91	85.03	84.53	76.48	85.41	85.54	85.91	85.16	85.54	85.91	81.89	84.78	85.03	84.28	83.15	82.01	84.65	87.17	85.66	94.47	95.09	94.59	94.97	-
50	70.06	70.82	72.83	72.83	75.72	75.72	71.45	81.38	81.01	82.77	78.62	79.62	75.85	81.38	80.76	80.76	76.35	75.47	80.13	83.69	83.27	80.88	81.76	75.09	79.25	82.52	81.76	80.50	74.97	81.89	80.13	81.13	80.38	80.38	81.38	78.24	81.51	81.64	81.26	81.13	76.48	80.88	81.38	80.25	80.50	81.01	80.76	81.01	79.62

Table S4 Differences in basic parameters between the 16S rRNA and *pyrG* (798 nt) gene sequences of *Bifidobacterium* subspecies: 1, *B. animalis* subsp. *animalis* / *lactis*; 2, *B. longum* subsp. *infantis* / *longum* / *suis*; 3, *B. pseudolongum* subsp. *globosum* / *pseudolongum*; 4, *B. thermacidophilum* subsp. *porcinum* / *thermacidophilum*.

	**Parameter**	**1**	**2**	**3**	**4**
**16S rRNA**	Lenght of sequences (nt)	1549	1485	1525	1485
	Mean sequence similarities (pairwise identity) (%)	99.0	99.4	99.1	99.0
	Identical sites (%)	99.0	99.1	99.1	98.5
	Mean number of nucleotide differences	16.0	8.70	13.0	14.7
pyrG	Mean sequence similarities (pairwise identity) (%)	97.0	97.4	95.7	97.6
	Identical sites (%)	97.0	95.0	95.7	96.5
	Mean number of nucleotide differences	24.0	21.0	34.0	19.0
